# Should administrative costs in health insurance be included in the risk-equalization? An analysis of five countries

**DOI:** 10.1007/s10198-022-01436-y

**Published:** 2022-02-07

**Authors:** Rudy Douven, Lukas Kauer, Sylvia Demme, Francesco Paolucci, Wynand van de Ven, Jürgen Wasem, Xiaoxi Zhao

**Affiliations:** 1 CPB Netherlands Bureau for Economic Policy Analysis, The Hague, The Netherlands; 2 CSS Institute for Empirical Health Economics, Lucerne, Switzerland; 3 University of Zurich, Zurich, Switzerland; 4 Federal Office for Social Security, Bonn, Germany; 5 University of Newcastle, Callaghan, Australia; 6 University of Bologna, Bologna, Italy; 7 Erasmus University Rotterdam, Rotterdam, The Netherlands; 8 University of Duisburg-Essen, Duisburg, Germany; 9 Boston University, Boston, USA; 10 University of Lucerne, Lucerne, Switzerland

**Keywords:** Risk-equalization, Risk-equalization payments, Administrative insurance costs, Loading fee, Medical claims, D40, G18, I11, I13, I18

## Abstract

Most countries that apply risk-equalization in their health insurance market(s) perform risk-equalization on medical claims but do not include other components of the insurance premium, such as administrative costs. Using fixed effects panel regressions from individual insurers in Australia, Germany, the Netherlands, Switzerland, and the US, we find evidence that health insurers with a high morbidity population on average have higher administrative costs. We argue that administrative costs should also be included in risk-equalization and we show that such equalization results in additional equalization payments nontrivial in size. Using examples from Germany and the US, we show how in practice policymakers can include administrative costs in risk-equalization. We are skeptical about applying risk-equalization to other components of the insurance premium, such as profits or costs related to solvency requirements of insurers.

## Introduction

Many countries with a competitive health insurance market have government regulations such as open enrollment for a basic benefit package, premium regulation, and risk-equalization. The goal of risk-equalization is to create a level playing field for health insurers and to prevent risk selection. The key element of ex-ante risk-equalization is to find the best prediction of an individual’s healthcare expenses for the new insurance year. After more than 30 years of research these predictions have been substantially improved [1, 2]. This raises the question: assuming we have a ‘perfect’ prediction of everyone’s future medical claims, is then the goal of risk-equalization achieved? The answer is no because in addition to medical claims enrollees also have to pay a loading fee. The loading fee is the excess of the premium above the expected medical claims to be paid by the insurer [3, p. 1237, 4, p. 181]. Depending on the characteristics of the insurance market, the share of the loading fee is about 5–20% of total premium payments. If insurers need to charge a higher loading fee for a high-risk than for a low-risk enrollee, there is still no level playing field and incentives for risk selection remain present.

In this paper, we focus on administrative health insurance costs which is a clearly demarcated cost category and, as we show, is the main cost component of the loading fee. In many countries, insurers have the obligation to report administrative costs annually. Administrative costs contain many different components often depending on the country regulations and type of insurance market [5, 6]. On one hand, some components are suitable for risk-equalization as they represent activities that an insurer has to undertake when providing health insurance. This holds, e.g., for administrative costs related to checking bills, fraud prevention, administrative contacts (phone, visits, email, etc.), costs for handling defaulters, purchasing healthcare, contracting healthcare providers, quality improvement, utilization management and the coordination of care might be lower for an insurer with a low-risk population than one with a high-risk population. On the other hand, not all administrative cost categories are suitable for risk-equalization. Examples are costs related to differences in administrative efficiency, for example due to (dis)economies of scale resulting from group size [4], unintended activities such as creaming, skimping or dumping of enrollees [7], marketing and advertising. These latter aspects should not be equalized because that would distort competition and create an unlevel playing field.

So far, however, the focus of risk-equalization by policymakers and in the literature has been primarily on the equalization of expenses for medical claims only. Often, the loading fee or administrative costs are overlooked. For example, in most papers on risk-equalization the loading fee is not even mentioned at all [1, 8]. A reason may be that in the past decades the efforts to improve the risk-equalization were primarily focused on the low-hanging fruit such as major morbidity indicators. Furthermore, administrative costs are not available at the enrollee level, as is the case with medical claims, making it difficult to show a causal relationship between the medical claims of an enrollee and the corresponding administrative costs.

Many countries with a risk-equalization system do not take administrative costs or the loading fee into account when applying risk-equalization. In this paper, we discuss three of these countries: Australia, the Netherlands and Switzerland, and two countries that do take administrative costs into account, Germany and the US. Germany found in an empirical analysis that around 50% of average administrative costs vary with medical claims and uses this number to risk-equalize insurers [9]. In the US Marketplaces, the rule was 100% until 2017. However, in 2018, the regulator substantially reduced the importance of administrative costs in risk-equalization, taking into account that (a proportion of) administrative costs do not vary with medical claims [10]. We discuss Germany and the US briefly in Sect. 4.2.

This paper is inspired by the experience in Germany [9] and, to the best of our knowledge, is the first paper that discusses to what extent the loading fee should be included in the risk-equalization. First, we study whether administrative costs vary with morbidity, measured by predicted expenses of medical claims. To obtain more causal evidence we test empirically the relationship for insurance markets in Australia, Germany, the Netherlands, Switzerland, and two markets in the US. We gathered several years of data per insurance market which allows us to use a fixed effect panel data regression, and to control for unobservable constant differences across insurers in a market. Although it is difficult to measure effects precisely, we find for most markets a positive correlation between administrative costs and the morbidity of an insurer’s population which enhances the premise of causality. Second, we discuss how administrative costs can be included by policymakers in a risk-equalization system and compare our method with the policy rules implemented by Germany and the US. Third, while this paper focusses on administrative costs, we also briefly discuss whether or not the residual part of the loading fee, such as costs related to profits or risk bearing, should be risk-equalized.

## Risk-equalization and administrative costs

Whether the administrative costs in health insurance should be included in the risk-equalization system depends on the goal of risk-equalization. This goal depends on the assumptions made about the health insurance market. In this paper, we assume a competitive health insurance market with regulation such as open enrollment for a basic benefit package and premium regulation in the form of community-rating (by class). The main challenge in a regulated competitive health insurance market is to avoid risk selection. We define risk selection as the actions by consumers and insurers to exploit unpriced risk heterogeneity and to break pooling arrangements [3, 11]. We assume that the goal of risk-equalization is to ensure that each applicant whom the insurer must accept represents an equal insurance risk for the insurer.^1^ This is the case if, and only if, the risk-equalization payment per enrollee provides a full compensation only for all enrollee-related risk characteristics that are not allowed to be used for premium rating. There should be no compensation for insurer-related characteristics such as an insurer’s efficiency, its market power, or the (dis)economies of scale resulting from its number of enrollees because that would distort the competition and create an unlevel playing field [8].

The main question for administrative costs is whether there is unpriced risk heterogeneity related to individual administrative costs (and loading fees). In contrast to medical claims, this question is extremely difficult to answer as administrative costs are not available at the individual level of the enrollee, but only at the insurer level. Moreover, most administrative cost components are strongly aggregated over various costs components making it even harder to attribute certain costs to individuals.

While it has been shown that high-risk enrollees have on average more administrative consumer contacts than low-risk enrollees, and thus are more costly for a health insurer [12], it is unknown to what extent there is unpriced risk heterogeneity related to an aggregate cost measure as total administrative costs. To obtain more insight into this problem, we study in this paper whether administrative insurance costs vary with medical claims at the insurer level. Thus, to what extent are administrative costs indeed higher for insurers with a high-risk population than for insurers with a low-risk population? In the next section, we will study empirically the relationship between administrative costs and an insurer’s population morbidity for six different insurance markets.

## Empirical analysis

If there is a causal effect of population morbidity on administrative costs, we expect to observe this relationship in many different insurance markets. Therefore, we selected six insurance markets that should satisfy the following criteria. First, there should exist a system of risk-equalization carried out by a regulator or government, as this allows us to obtain an exogenously determined indicator for population morbidity of each insurer, which in the remaining part of the paper we call the insurer’s risk-score. Second, there should be a sufficient number of insurers in the market to obtain enough cross-sectional variation. For example, we excluded Belgium, Chile, Ireland and Israel because each of these countries has a limited number of insurers. Moreover, there should be multiple years of data available to perform a panel regression with fixed insurer effects. This is important as administrative costs may differ in many unobservable dimensions across insurers, such as differences in efficiency (capital and personnel), profit requirements (for-profit, not-for-profit or social insurer), market power, size, providing other type of insurance activities, different benefit packages, etc. The fixed-effects regression will eliminate constant unobserved differences across insurers. We gathered data for various years in six markets of five countries: Australia, Germany, the Netherlands, Switzerland and two insurance markets in the US, the small group and the individual market (the so-called US Marketplaces). The data are obtained from insurer reports that are publicly available or assembled by the country regulator.^2^ For each insurance market, a description of the market and data is provided in “Appendices 1–6”. Premium revenues, medical claims and total administrative costs are demarcated cost categories that are available in all annual insurer reports.

**Fig. 1:**
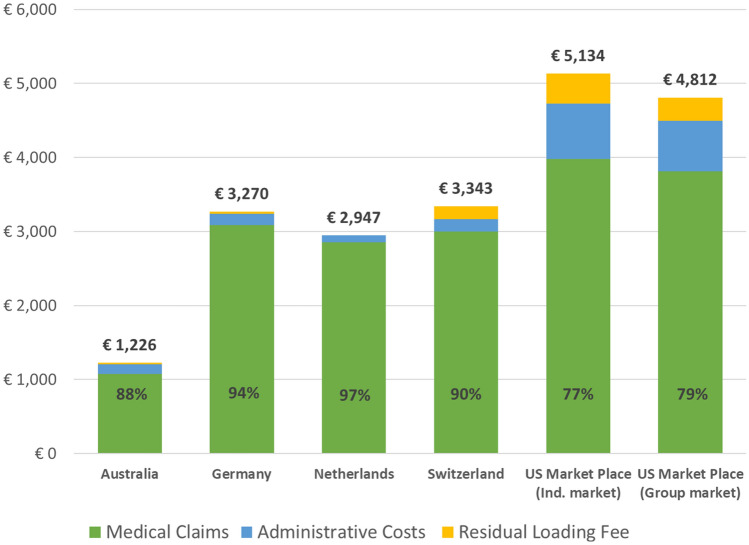
Mean premium paid per person per life year and its major components (to facilitate comparison across countries all numbers are in euros, 2019). The numbers above the bar represent the average premium in the insurance market in 2019 for the basic benefit package. The premiums are in euros using the following exchange rates (April 10, 2021): 1 Euro = 1.56 Australian Dollar = 1.19 US Dollar = 1.10 Swiss Franc. The premium includes subsidized premium payments and risk-adjusted payments insurers receive from (or pay to) the regulator. Medical claims represent payments from insurers to health care providers. Payments of claims by consumers related to cost-sharing arrangements are not included in these payments. Administrative costs are total administrative costs. The residual loading fee is computed by subtracting medical claims and administrative costs from the premium. The percentage numbers in the bars represent the share of average costs for medical claims in the premium. One minus this fraction represents the share of the loading fee (administrative costs plus the residual loading fee) in the premium. All numbers are obtained from annual reports of health insurers or assembled by the country regulator. For the numbers of health insurers included see Table 1. For more information, we refer to “Appendices 1–6”

To obtain an idea about the different components of the premium, we show in Fig. 1 the mean premium per enrollee per life year for each market in 2019, divided into three components; medical claims, administrative costs and a residual loading fee component.^3^ The sum of this residual component and administrative costs are often denoted as the loading fee [13]. To ease comparability across the countries we converted all amounts into Euros. Figure 1 shows some interesting differences across the six insurance markets. Note that the numbers in the figure can only be broadly compared across markets as markets may differ in various ways, such as in the size of the basic benefit package, cost-sharing arrangements, contracting and efficiency activities by insurers, economies of scope due to activity in other insurance markets, insurance regulations, type and size of insurers in the market, culture, etc. Costs for medical claims and residual loading fees are clearly highest for enrollees in the two US-markets, with mean administrative costs accounting for about 14% of the premium and the loading fee for more than 20%.^4^ These costs are substantially lower for the other four countries; administrative costs (loading fees) range from 3% (3%) of premium payments in the Netherlands to 11% (12%) in Australia. In the Netherlands, we find even slightly negative residual loading fees because some insurers in 2019 used their excessive reserves to lower their annual premiums.

**Table 1:** Summary statistics of health insurance market per country

Country	Australia	Germany	Netherlands	Switzerland	US market place	US market place
Type of insurance	Supplementary insurance	Basic insurance	Basic insurance	Basic insurance	Basic insurance (individual market)	Basic insurance (small group market)
Years	2010–2019	2014–2019	2014–2019	2010–2019	2017–2019	2017–2019
# Observations (all years)	208	673	58	593	911	941
# Insurers (range per year)	19–23	104–123	9–10	51–81	284–339	284–342
Level of observation: Insurer carrier/holding	Carrier	Carrier	Holding	Carrier	Carrier	Carrier
Mean population size of insurers	572,378 (1,015,647)	641,547 (1,566,407)	1,375,421 (1,403,130)	137,858 (207,596)	48,524 (96,092)	40,702 (81,849)
Mean risk-score of insurers	0.98 (0.11)	0.90 (0.16)	0.93 (0.11)	1.00 (0.26)	1.01 (0.19)	0.99 (0.07)
Currency	Australian Dollars	Euros	Euros	Swiss Francs	US dollars	US dollars
Mean administrative costs per person	167.6 (51.9)	146.1 (30.3)	102.0 (22.8)	164.6 (67.7)	849.4 (432.3)	824.8 (245.0)
Mean premium per person	1559.2 (389.1)	2949.1 (2226.8)	2684.9 (381.6)	3090.8 (583.7)	5530.0 (2108.0)	5462.8 (912.2)
Mean medical claims per person	1357.0 (280.8)	2789.7 (506.8)	2627.3 (401.2)	2833.0 (1194.7)	4502.8 (1912.5)	4318.1 (829.3)

The table is constructed from annual insurer reports in each country for each year. The risk-score for insurer $i$ is computed by ($\overline{{{\text{mc}}}} + {\text{ra}}\left( i \right) - \overline{{{\text{ra}}}} )/\overline{{{\text{mc}}}}$, where $\overline{{{\text{mc}}}}$ is a scaling parameter representing the average amount of medical claims in the market per life year. ${\text{ra}}\left( i \right)$ is the average risk adjusted payment per person that insurer $i$ receives from the regulator and $\overline{{{\text{ra}}}}$ is the average risk adjustment payment per insurer per person in the total market. The mean risk-scores are not equal to 1 as we have not weighted them with the population size of an insurer. A mean risk-score below 1 in a market indicates that large insurers have a relatively more morbid population. For an explanation of per person per life year administrative costs, premium and medical claims, see note below Fig. 1. Standard deviations in parentheses.

Table 1 provides the summary statistics of the data we use in our panel regressions. Again, we refer to “Appendices 1–6” for a detailed description of each market. We have five markets that offer basic insurance and one market, Australia, that offers supplementary insurance. The number of insurers and the population size of insurers vary across markets. For all countries, the information is at the level of the insurance carrier, except for the Netherlands where we could obtain data at the holding (several carriers under one roof) level only. This is reflected in Table 1, where Dutch holdings have on average more than one million enrollees. The insurer carriers in the two US markets are smallest in size with on average less than 50,000 enrollees per insurer. Insurer size seems also to be (partly) reflected in the administrative costs as in general, insurers with more enrollees have fewer administrative costs per enrollee due to economies of scale.

To study whether administrative costs vary with expected medical claims we constructed the risk-score for each insurer, using the reported risk-equalization payments that insurers receive from, or have to pay to, the regulator in the market. To make the risk-scores broadly comparable across countries we performed a similar strategy among countries and scaled the payments with average medical claims in a market to obtain average risk-scores around 1 (see notes below Table 1). Note that the rules for applying risk-equalization are determined in all markets ex-ante by the regulator and, therefore, the risk-scores can be considered as exogenous with respect to the medical claims in the new insurance year. However, the constructed risk-scores may contain potential measurement errors.^5^ We discuss this point more extensively in “Appendix 1”, where we also run regressions with an ex-post risk-score variable that we constructed with medical claims in the current year.

**Fig. 2:**
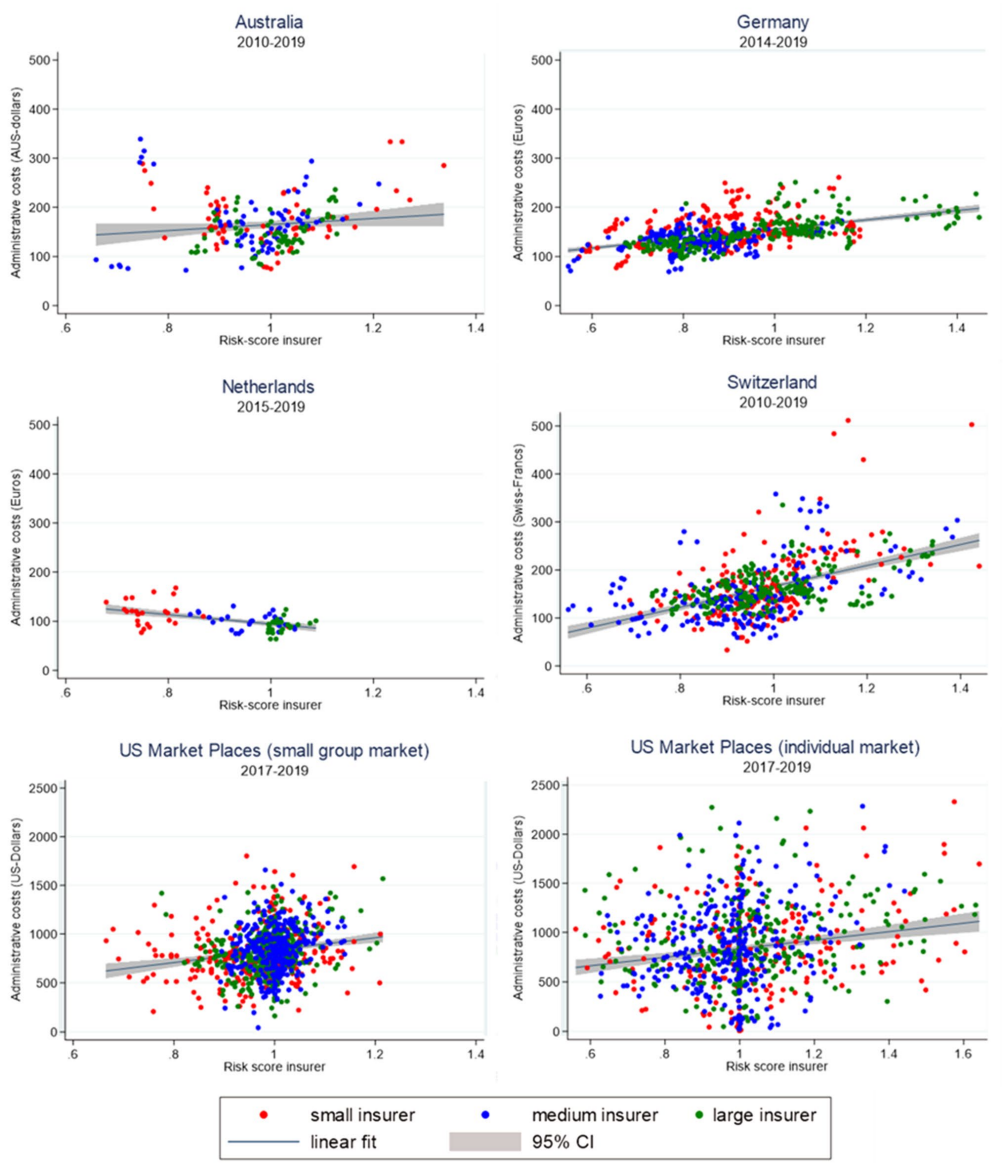
Insurer’s administrative costs (per person per life year) and the risk-score. To obtain comparability across countries the range of the x-axis is the same for all countries. Only for the US Marketplace (individual market), we extended this range to 1.6, and still 14 observations fall outside this (extended) range. In Switzerland, all observations of one insurer are excluded from the figure because they fall outside the range. A linear trend is plotted in the figure with a 95% confidence interval (95% CI)

Figure 2 shows the correlation in the raw data between mean administrative costs per life year and the risk-score for each insurer in the six markets. Because economies of scale play an important role in administrative costs [13], we divided insurers in each market into three equally sized groups: small (0–33 percentile), medium (34–66 percentile) and large (67 + percentile) insurers, each group represented by a different color in the figure. Five of the six graphs show a positive correlation between administrative costs and an insurer’s risk-score. The insurer’s risk-scores for most markets range between 0.5 and 1.5, only for the individual market in the US we find risk-scores above 1.5 for some insurers. For Germany and Switzerland, and to a lesser extent Australia, the observations are about equally distributed on the x-axis. Also, small, medium, and large insurers are distributed over the whole x-axis which indicates that there is sufficient variation to obtain plausible estimates. For the Netherlands, we have limited variation and find a negative relationship. However, this relationship seems to be driven by economies of scale as population size is strongly positively correlated with the risk-score. This relationship is more clearly visible in the Netherlands, and less so in other markets, because there are only a few Dutch insurers (holdings) in the market and the differences in the market share between the largest insurer (about 27%) and smallest insurer (around 0.5%) is much bigger in the Netherlands than in other countries, which emphasizes the economies of scale effects. Therefore, it is important to control for population size in our estimations. The data in the US small group market are more concentrated around 1 on the x-axis then in the US individual market. Both US markets show substantial variation in administrative costs on the y-axis which suggests that the positive correlations are less clearly present in the data. Note that due to the high administrative costs in the US markets, the scale of the y-axis for the two US markets is a factor five of the other four markets.

Table 2 shows the fixed effects estimates for each market of the following panel regression:

1 $${\text{adm}}_{it} = \beta_{0} + \beta_{1} {\text{riskscore}}_{it} + \beta_{2} {\text{popsize}}_{it} + \beta_{3} \left( {\frac{1}{{ {\text{popsize}}_{it} }}} \right) + \alpha_{i} + \gamma_{t} + \varepsilon_{it} ,$$

where subscript $i$ represents the insurer and $t$ the insurance year. The dependent variable is ${\text{adm}}_{it}$, mean administrative costs per life year and ${\text{riskscore}}_{it}$ is the insurer’s risk-score. $\alpha_{i}$ denotes insurer fixed effects and $\gamma_{t} { }$ year fixed effects. Although the insurer fixed effects control for the average market size of an insurer, we also included an insurer’s population size, ${\text{popsize}}_{it} ,$ and the inverse of population size to capture possible non-linear time varying aspects. $\varepsilon_{it}$ represents the error term. $\beta_{1}$ is our coefficient of interest as it measures the effect of an increase in the risk score on the administrative costs. Note that we cluster standard errors at the insurer’s level.

**Table 2:** Fixed effect estimates of $\beta_{1}$: the effect of an insurer’s risk-score on administrative costs

Dependent variable: administrative costs						
Country	Australia	Germany	Netherlands	Switzerland	US (individ.)	US (group)
*Years*	2010–2019	2014–2019	2014–2019	2010–2019	2017–2019	2017–2019
*Controls*						
Risk-score	313.8**	86.0*	70.8	197.8**	509.6	471.4**
	(85.8)	(33.9)	(83.9)	(48.7)	(300.7)	(192.1)
Population size	Yes	Yes	Yes	Yes	Yes	Yes
Year fixed effects	Yes	Yes	Yes	Yes	Yes	Yes
Insurer fixed effects	Yes	Yes	Yes	Yes	Yes	Yes
# Observations	208	673	58	593	911	941
R-squared	0.91	0.89	0.88	0.87	0.81	0.84

**, *Significance at the 0.01 and 0.05 level. Clustered standard errors in parentheses. We refer to “Appendices 1–6” for the specific results.

We find a positive coefficient for the risk-score for all insurance markets. For four markets, we find a statistically significant positive effect, suggesting that insurers with a high-risk population have higher administrative costs. The negative correlation in the raw data for the Netherlands (see Fig. 2) becomes positive after controlling for market share, but the effect is statistically insignificant.^6^ The effects are substantial considering that the risk-scores in most markets range between 0.5 and 1.5. This implies that the estimates in Table 2 can be roughly interpreted as the difference in administrative costs between an insurer with the highest and the lowest risk population. For example, in Germany the difference in risk-scores between the lowest and highest insurer is about 0.8 amounting to a difference in administrative costs of 0.8*86.0 = 69 euros per person per life year.

**Fig. 3:**
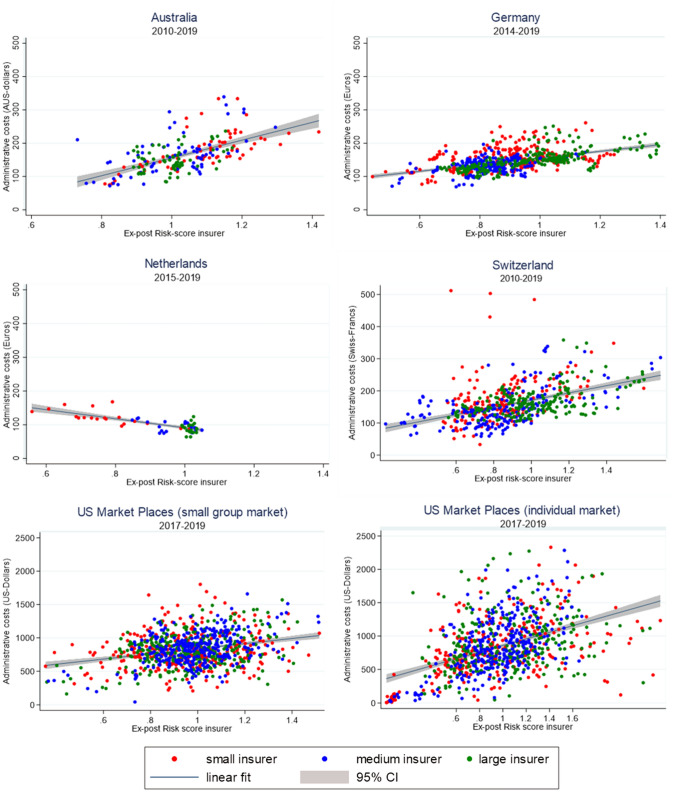
Administrative costs (per person per life year) and the ex-post risk-score. To obtain comparability across countries and to ease the comparison with Fig. 2 we also kept the numbers on the x-axis the same as in Fig. 2 for all countries. From this graph follows that the variation of the ex-post risk score is considerably larger for Switzerland and the US Marketplaces (both markets) (see also Table 3.)

The results in Table 2 are for our most preferred specification. In “Appendix 1”, we test the robustness of our results and run our panel regressions also with an ex-post risk-score, constructed by dividing an insurer’s average expenses of medical claims per life year by the market average. This ex-post risk-score introduces endogeneity problems as it is determined in the same year as administrative costs. Figure 3 in “Appendix 1” suggest that the correlation between the ex-post risk-score and administrative costs are about equal for Germany, the Netherlands and Switzerland than in Fig. 1, but the positive correlation becomes larger for Australia and the US. The fixed effect estimates that follow from the regressions with the ex-post risk-score are somewhat smaller and statistically insignificant for Australia and the US, which suggest that it is difficult to measure the effects precisely for these countries. The estimates remain positive and statistically significant for Germany and Switzerland.

We conclude that there is evidence for a positive causal effect of an insurer’s population morbidity on its administrative costs. The estimated effect is relatively stable for Germany and Switzerland. However, it is unclear how large the size of the effect is for the US, Australia and the Netherlands as the estimated effects are surrounded with more uncertainty. The likely reason for the US is that there is extensive variation in administrative costs across insurers and the ex-ante risk-score may contain more potential measurement errors than in other countries (see “Appendix 1”). In Australia and the Netherlands, the annual number of insurers in the market is relatively small which complicates measuring the effects precisely.

## How can administrative costs be included in the risk-equalization system?

When applying risk-equalization most regulators (or governments) use individual annual medical claims, often from the total population, and regress these claims on individual consumer characteristics [2, 8]. However, administrative costs are not available for individual consumers and are only available at the insurer level. Thus, a different approach is needed.

We first show a methodology for risk-equalizing administrative costs and then discuss the policy rules adopted in Germany and the US.^7^ Our methodology uses the estimated effects of an insurer’s risk-score on the average administrative costs per enrollee (see Table 2).

As in the previous section we denote the average risk-score for an insurer $i$ with ${\text{riskscore}}_{i}$, where the average is taken over the predicted risk-scores of all enrollees of insurer $i$. The predicted risk-score of an enrollee is based on the predicted medical claims of an enrollee, based on for example demographics and diagnoses of an enrollee. We scale the average predicted risk-score of an enrollee to 1. Thus, an insurer with a ${\text{riskscore}}_{i}$ > ( <) 1 will have higher (lower) predicted medical claims per enrollee in the new insurance year than an insurer with ${\text{riskscore}}_{i}$ = 1.

Most countries risk-equalize only medical claims, i.e., the regulator predicts $\widehat{{{\text{MC}}}}$, the predicted average medical claim per enrollee in the market. $\widehat{{{\text{MC}}}}*{\text{riskscore}}_{i}$ then reflects the predicted amount of medical claims for the average enrollee of insurer $i$.^8^ Note that in these countries the regulator does not predict the loading fee or administrative costs and thus both components do not vary with medical claims in risk-equalization.

### Formula for risk-equalizing administrative costs

A method to risk-equalize administrative costs that vary with the insurer’s risk-score, is to use the regression results in Table 2. From Eq. (1) follows that $\widehat{{{\text{adm}}}}_{it} = \hat{\delta }_{it} + \hat{\beta }_{1} {\text{riskscore}}_{it}$, where $\hat{\delta }_{it}$ represents the part that is independent of the risk-score and, thus, does not vary with medical claims.^9^ We define $\widehat{{{\text{ADM}}}}_{t}$ as the mean predicted administrative costs per life year, with the average taken over all insurers. Since the average annual risk-score of all insurers is constructed to be 1, $\widehat{{{\text{ADM}}}}_{t} = \hat{\delta }_{t} + \hat{\beta }_{1} ,$ with $\hat{\delta }_{t}$ the average of $\hat{\delta }_{it}$ taken over all insurers. Because $\hat{\delta }_{it}$, the first component of $\widehat{{{\text{adm}}}}_{it}$, contains insurer-specific aspects (i.e., the insurer fixed effect and its population size) that the regulator (most likely) does not want to equalize, the predicted administrative costs of insurer $i$ that are acceptable to be equalized under a zero-sum equalization can be specified as $\widehat{{{\text{adm}}}}_{it} = \hat{\delta }_{t} + \hat{\beta }_{1} {\text{riskscore}}_{i}$. The equalization payment for insurer $i$ then equals $n_{i}$ ($\widehat{{{\text{adm}}}}$_*it*_*–*$\widehat{{{\text{ADM}}_{{\text{t}}} }}$) = $n_{i} \hat{\beta }_{1} ({\text{riskscore}}_{i} - 1),$ with ${\text{n}}_{i}$ equal to the number of enrollees of insurer $i$. For example, an insurer with a risk-score 0.7 has to pay an equalization payment of 0.3*$\hat{\beta }_{1}$ per enrollee, while an insurer with a risk-score of 1.3 receives 0.3*$\hat{\beta }_{1}$ per enrollee. In the six insurance markets 0.6*$\hat{\beta }_{1}$ ranges between about 40 euro and 300 euro (see Table 2), which implies that risk-equalization of administrative costs that vary with the insurer’s risk-score, is nontrivial. Because an insurer’s risk-score may change after enrollees have switched insurers during the open enrollment period, it should be calculated after the open enrollment period. This equalization of administrative costs can be done in addition to the equalization of medical claims, or it can be combined with it.

The second component of $\widehat{{{\text{ADM}}}}_{t} ,$ i.e., $\hat{\beta }_{1}$, depends on the risk-scores and therefore represents the part of $\widehat{{{\text{ADM}}}}_{t}$ that is risk-equalized. Thus, the percentage of administrative costs that is used for risk-equalization equals $\frac{{\hat{\beta }_{1} }}{{\widehat{{{\text{ADM}}}}}}_{t} {*}100{\text{\% }}$. Using the mean administrative costs over all relevant years per insurance market from Table 1 and the estimates $\hat{\beta }_{1}$ from Table 2 yields the following back-of-the-envelope percentages for Australia: 187%, Germany: 59%, the Netherlands: 69%, Switzerland: 120%, US (individual market): 61%, US (group market): 57%.^10^

### Germany and the US marketplaces

Germany is, as far as we know, the only country that uses an empirical prediction of administrative costs [9, 14]. Drösler and co-authors find a positive effect of the risk-score on administrative costs which resulted in the policy rule that 50% of the administrative costs should be risk-equalized. The 50% rule is in line with our results where we find 59% (see above). Thus, while Germany includes administrative costs, they do not include the residual loading fee in risk-equalization.

Before 2018, in the US marketplace the risk-equalization payments for insurer $i$ were based on the rule $\overline{P}*{\text{riskscore}}_{i}$, where $\overline{P}$ is the average premium in the market. So, the US applied the rule that the loading fee (administrative costs and residual loading fee) should be equalized for 100%. As of 2018, the US substantially reduced the importance of the loading fee in the risk-equalization. In the formula for risk-equalization, the average premium in the market is reduced by a fixed rate of 14% with the argument that this reduction reflects (the proportion of) administrative costs that do not vary with medical claims [10].^11^ Thus, the new rule implies that risk-equalization will be based on 0.86 $*\overline{P}*{\text{riskscore}}_{i}$. Our estimation results for the US are too imprecise to judge whether the new rule should be preferred over the old rule. The estimations of the risk-score indicate that the risk-equalization should be based on 0.94 $*\overline{P}*{\text{riskscore}}_{i}$ which implies lower equalization payments in the US Marketplaces for administrative costs than the old rule, but higher equalization payments than then new rule. The estimation results for the ex-post risk-score in “Appendix 1” implies that risk-equalization should be based on 0.85 $*\overline{P}*{\text{riskscore}}_{i}$ which is in line with the new rule.^12^

## Discussion

The main message of our analyses is that administrative costs should be taken into account when applying risk-equalization. In this paper, we show how this can be done by relating the mean administrative costs per enrollee to an insurer’s risk-score. However, other routes are possible as well. The most obvious approach is that the regulator requires insurers to decompose their administrative costs into several components. For each of these components the regulator can determine to what extent they are eligible for risk-equalization. For example, one component could be the administrative costs for enrollees that cannot pay their deductible or out-of-pocket premiums and end up in payment arrears. In case of an insurance mandate where insurers cannot terminate coverage, insurers have often to undertake costly activities to collect this money. The probability of ending up in payment arrears is likely to be unevenly distributed across individuals.

Our approach, i.e., considering the mean administrative costs per enrollee and relate this to an insurer’s risk-score, should be seen as a practical approximation.^13^ This approach will only work if there is enough variation in various dimensions in the data to credibly control for all types of insurance aspects. If the number of insurers is relatively small or variables as risk-scores, population size and other administrative activities are unevenly distributed, it is difficult to obtain credible estimates. Moreover, if the risk-score follows from imperfect risk-equalization of medical claims, then relating the risk-score to administrative costs will result in biased estimates (see also “Appendix 1”). Thus, applying a simple rule, like the US and Germany do, seems to be a practical solution to a complicated problem. It is transparent and to be preferred over applying no rule at all. The exact percentage can be calculated as we do in this paper. However, our computations can be improved if there is better and more precise information available about components of (expected) administrative costs, risk-scores, etc.

We are sceptical about following a similar strategy for the residual loading fee, i.e., correlating the loading fee with an insurer’s risk-score.^14^ A first reason is that it is a priori not clear whether the residual loading fee contains many categories that are suitable for risk-equalization. It is doubtful whether a regulator should want to equalize for components of the residual loading fee such as profits related to market power, profit windfalls or shortfalls (that may be correlated across insurers), or solvency requirements that insurers have to meet. However, the cost of risk-bearing could be a potential candidate for risk-equalization as the expected variation in medical claims is often larger for high-risk than for low-risk individuals. All else equal, risk averse insurers might want to charge a higher risk-premium for high-risk individuals. A second reason is that endogeneity problems might also play a role. For example, consider a case of perfect competition (where insurers have to charge the same premium for the basic benefit package) and imperfect risk-equalization of medical claims, then insurers who are undercompensated will face lower profits. A third reason is that several components are not clearly demarcated, also because they often contain transfers from previous years, making it difficult to precisely measure the costs of single components or the total residual loading fee in a year.

Finally, there might be potential disadvantages of including administrative costs in risk-equalization as it might incentivize cost inflation. For example, insurers might see possibilities to game the risk-equalization system by increasing their administrative activities or by shifting administrative costs between several cost components, such as shifting administrative costs related to supplementary insurance to basic insurance. These potential disadvantages are likely to be relevant in concentrated markets where insurers have relatively large market shares, such as for example in the Netherlands and Australia, as the marginal returns from increasing administrative costs increases with the market share. To prevent gaming activities, policymakers should clearly define the various administrative cost categories so that they can be properly monitored. An extreme option to prevent cost inflation is putting a constraint on the size of administrative costs, as is done in the US. In the Affordable Care Act insurers must remit a rebate if the loading fee is larger than 20% of the premium. Another option is to define the total amount of administrative costs on a percentage of medical costs only.

## Conclusion

Many countries with a risk-equalization system in health insurance risk-equalize only medical claims of enrollees. We argue in this paper that components of the loading fee should be considered for risk-equalization as well, with the loading fee defined as the excess of the premium above the expected medical claims to be paid by the insurer. The reason is that enrollee characteristics, such as being a high or low-risk enrollee, likely have a causal impact on the loading fee. We show for six insurance markets in five countries that an insurer’s administrative costs, which we show is the major component of the loading fee, is positively correlated with an insurer’s risk-score as an indicator for the morbidity of the insurer’s population. We show how in practice administrative costs can be included in risk-equalization in a simple way and we show that this results in additional equalization payments nontrivial in size. We discuss the examples of Germany and the US marketplaces. The policy rule in Germany that 50% of the administrative costs should be risk-equalized, is consistent with our empirical findings. For the US, our empirical results are too imprecise to judge the current US-rule and more research is needed.

As far as we know, our paper is the first to address this important issue. There are many channels for future research. A first channel would be to obtain better knowledge of why administrative costs may differ across enrollees. For example, Douven and Kauer [12] show that high-risk enrollees cause more consumer contacts then low-risk enrollees. But other interesting administrative differences could occur for handling defaulters, purchasing healthcare, contracting healthcare providers, utilization management, etc. A second channel is to improve our regressions for individual markets by obtaining better data about administrative cost components and risk-scores. Policymakers could require insurers to decompose administrative costs into different components, components that are suitable and not suitable for risk-equalization. Also, the measurement of an insurer’s risk-score could be more precisely computed by taking specific characteristics of insurance markets better into account. A third channel to consider is that administrative costs may depend on characteristics not necessarily related to health risks. For example, insurers may face higher administrative costs for empowered consumers or consumers who have a larger chance to end up in payment arrears.

We are more skeptical about risk-equalizing the residual loading fee, i.e., the difference between the loading fee and administrative costs. The residual loading fee is not a clear demarcated cost category and contains a lot of components that may not be suitable for risk-equalization. More research is needed whether some specific cost components of the loading fee are suitable for risk-equalization.

## Data Availability

The data are publicly available for the countries Australia, the Netherlands, Switzerland and the US. These data can be retrieved from the corresponding author upon request. The data for Germany are proprietary.

## Appendix 1: Robustness analysis

In countries with regulated competition, health insurers are mandated to report the payments they receive from, or have to pay to, the regulator, due to risk-equalization. From this payment, we calculate $ra\left( i \right)$, the average risk adjusted payment per person that insurer $i$ receives from or pays to the regulator and $\overline{{{\text{ra}}}}$ is the average risk adjustment transfer per insurer per person in the total market. To obtain comparability across countries, we computed the risk-score for insurer $i$ in each country in the same way by ($\overline{{{\text{mc}}}} + {\text{ra}}\left( i \right) - \overline{{{\text{ra}}}} )/\overline{{{\text{mc}}}}$, where $\overline{{{\text{mc}}}}$ is a scaling parameter representing the average amount of medical claims in the market per life year. This measure may contain errors. First, the payment ${\text{ra}}\left( i \right)$, which is often based on a regression to determine individual expected medical claims of enrollees, may be imprecisely measured, for example because the risk-adjusters applied in the regression do not sufficiently control for unpriced risk heterogeneity. Another potential measurement error may occur because we assume that ${\text{ra}}\left( i \right)$ can be determined by dividing the payment that insurers receive by the number of enrollees. Australia allows premium discounts for young adults (19–28 years old), in Germany children and non-working spouses pay no own contributions, in the Netherlands children pay no premium, in Switzerland, children and younger adults pay a lower premium than older adults, in the US (small group and individual market) the premium may differ by age group, family size, geographical region, and smoking status. A measurement error in our constructed risk-score may occur when there is skewness in the distribution for these specific groups of enrollees across insurers. Unfortunately, this information about specific groups of enrollees within insurers is not consistently available in the annual insurer reports. For all these reasons, we perform a robustness analysis in this section.

Another way to calculate risk-scores is to use medical claims in the current insurance year. This ex-post risk-score ${\text{mc}}\left( i \right)/\overline{{{\text{mc}}}}$ represents the average medical claims per enrollee for insurer $i$ divided by the average medical claims per enrollee in the total market. This is an imperfect measure as it does not measure expected but actual medical claims and thus suffers from endogeneity problems. Also, actual medical claims may not be measured precisely as for some countries out-of-pocket payments are not included. In Figure 3, we reproduce Fig. 2 in the main text replacing the risk-score by the ex-post risk score. The positive relationship between the ex-post risk score and administrative costs seems to be even more pronounced in this graph, especially for Australia and the two US markets. The means and standard deviations of the risk-score and ex-post risk-score are presented in Table 3. The variation of the ex-post risk-score is somewhat larger for Switzerland and the two US markets. In the lower panel of Table 3, we compare the estimation results for both risk-scores. We find some differences across countries but in general the estimates remain positive. Only for the Netherlands we find an insignificant negative estimate which might be related to the low number of observations. Contrary to the results in Figure 3, we find in our fixed effects regressions somewhat smaller positive and insignificant estimates for Australia and the US. For Germany and Switzerland, the results remain positive and significant. In the bottom part of Table 3, we performed the same regression but excluded insurers with fewer than 10,000 enrollees from the sample (note that the estimations for the Netherlands are not affected as all insurers had more than 10,000 enrollees). The results turn out to be quite robust to excluding these insurers from the sample.

**Table 3:** Estimates for different risk-scores and exclusion of small insurers from sample

Country	Australia	Germany	Netherlands	Switzerland	US (individ.)	US (group)
Years	2010–2019	2014–2019	2014–2019	2010–2019	2017–2019	2017–2019
MEAN RISK-SCORE						
Ex-ante risk-score	0.98	0.90	0.93	1.00	1.01	0.99
of insurers	[0.11]	[0.16]	[0.11]	[0.26]	[0.19]	[0.07]
Ex-post risk-score	1.03	0.90	0.92	0.94	0.97	0.97
of insurers	[0.14]	[0.16]	[0.13]	[0.35]	[0.41]	[0.18]
REGRESSIONS						
Dependent variable: administrative costs						
*Regression in main text*						
Ex-ante risk-score	313.8**	86.0*	70.8	197.8**	509.6	471.4**
	(85.8)	(33.9)	(83.9)	(48.7)	(300.7)	(192.1)
# Observations	208	673	58	593	911	941
R-squared	0.91	0.89	0.88	0.87	0.81	0.84
*Regression with ex-post risk-score*						
Ex-post risk-score = ${\text{mc}}\left( i \right)/\overline{{{\text{mc}}}}$	42.9	82.2*	− 41.8	91.2**	21.9	2.8
	(28.0)	(34.1)	(47.6)	(37.9)	(128.4)	(118.4)
# Observations	208	673	58	593	911	941
R-squared	0.88	0.89	0.88	0.86	0.80	0.84
*Regression in main text with insurers with population size* > *10,000 only*						
Ex-ante risk-score	285.4**	76.2*	70.8	219.0**	752.5	393.6
	(106.1)	(42.2)	(83.9)	(45.0)	(282.8)	(334.2)
# Observations	197	605	58	393	586	600
R-squared	0.91	0.89	0.88	0.87	0.80	0.87
*Regression with ex-post risk-score and with insurers with population size* > *10,000 only*						
Ex-post risk-score = ${\text{mc}}\left( i \right)/\overline{{{\text{mc}}}}$	82.9	87.0*	− 41.8	138.3**	24.1	110.8
	(57.8)	(42.9)	(47.6)	(26.9)	(177.5)	(223.7)
# Observations	197	605	58	393	586	600
R-squared	0.88	0.89	0.88	0.86	0.80	0.70

**, *Significance at the 0.01 and 0.05 level. Standard deviations in brackets and clustered standard errors in parentheses. All regressions include fixed insurer and year effects and two variables for population size. We refer to “Appendix 2–6” for the specific results per country. Note that for the Netherlands there are no insurer holdings with a population size smaller than 10,000 and, therefore, we have no changes in the column of the Netherlands.

## Appendix 2: Australia

Australia’s healthcare system is characterized by a strong public–private mix. It is organized through a mandatory, universal tax financed scheme, Medicare, and on top, individuals may purchase duplicative coverage (e.g., hospital treatment) and supplementary insurance (e.g., general treatment) through private insurance. This summary is based on the supplementary private health insurance (PHI) as that is where risk-equalization is present.

In 2007, the Private Health Insurance Act established the current regulations pertaining PHI. Several components of a managed competition type system are present. The “principle of community rating” practically implies premiums do not vary according to risk characteristics of the enrollees (with exception of allowed discounts for those 18–29), and there is open enrollment. Regulations in place provide ‘sticks and carrots’ to encourage enrollment, such as the lifetime health cover ‘loading’ (a penalty for taking up insurance after 31), Medicare levy surcharge (a levy imposed for high income earned who do not take up insurance), and premium reduction scheme through subsidies (or tax offset) to hospital and general treatment policies which currently amount to 6.3 billion AUD to increase affordability. Most recently, reforms efforts of April 2020 into improving transparency in the market established product tiers (e.g., basic, bronze, silver and gold) for hospital coverage composed by clinical categories, where the basic benefit package is contained in the basic tier.

Despite a majority of insurers being not-for-profit, for-profit insurers cover a significant proportion of the policies (e.g., BUPA and Medibank—concentrate more than 50% of enrollment).

The Australian Risk Equalisation Trust Fund began on 1 April 2007 and succeeds a similar scheme, the Reinsurance Trust Fund, and currently uses only age and predefined percentages as weights in the equalization (so called, claims equalization) [15]. Eligible benefits are composed by hospital benefits, hospital substitute benefits, and chronic disease management program benefits (CDMP). Embedded in the formula, a payment scheme is the compensation for 82% of those benefits that exceed a threshold of 50,000 AUD (discounting payments from the age-based formula).

The data we use in our analysis are obtained from the Australian Prudential Regulation Authority (APRA), and historical data from the Private Health Insurance Administration Council (PHIAC), the previous regulator of the industry whose functions were transferred to APRA. Information from 23 insurers in 2010, 22 in 2011–2012, 21 in 2013–2014, 20 in 2015–2018 and 19 in 2019 are used and come from two sources: The Operations of Private Health Insurers Annual Reports, which contain information according to the Prudential Supervision Act 2015, regarding financial performance, financial position, membership, revenues and expenditures, and in particular management expenses which constitute operating expenses incurred in the course of normal fund operations (e.g., salaries, commission, rent), and the Private Health Insurance Risk Equalisation Financial Year Results, which contains information on the transfers to/from insurers in the Risk Equalisation Trust Fund.

## Appendix 3: Germany

The following summary of the German health insurance system draws from Wasem et al. [14]. Germany has a two-tiered system, with 90% of the population being insured in one of (at present) about 104 social health insurance institutions, so called “sickness funds”. A third of them are company-based sickness funds which can only be chosen by the employees of the company. The remaining 10% of the population have their coverage primarily with private health insurance companies. Since 2009, it is mandatory in Germany to be insured in one of the two health insurance systems. In this paper, we only deal with social health insurance.

Sickness funds are obligated to cover all medical services included in the benefits package as specified on the national level. In addition, they can offer supplementary benefits, such as more generous services with regard to home nursing or non-prescription drugs. These additional benefits add up to less than 1% of total spending under sickness fund insurance. Benefits are delivered primarily in kind, with members showing their health insurance card to the health care provider. Insured have, by and large, free choice among all providers that are part of the collective contract system. Individual sickness funds have only limited discretion with regard to the contractual relations to providers as contracts between the national or state level associations of sickness funds with corresponding associations of providers prevail.

Social health insurance is financed primarily by income-related contributions up to a certain ceiling. Employers cover half of the contributions of their employees; unemployment offices pay contributions for the unemployed. Children and non-working spouses are covered without own contributions. All contribution payments are collected by a “Central Health Fund”, which pays risk-adjusted subsidies to the individual sickness funds according to the risk structure of their insured. The risk-adjusted subsidies cover on average 93% of the expenditures. For the remaining part, individual sickness funds have to calculate fund specific “additional contributions”, the differences of which between the sickness funds are a major driver for competition. The additional contributions raised by the sickness funds are paid by all members independently of their individual risk. Insured can switch sickness funds after a minimum of 12 months of membership; sickness funds are not allowed to reject anyone eligible for insurance.

Risk adjustment via the subsidies from the central health fund has increasingly become more sophisticated. During the period of analyses in this paper, it consisted of variables for age and gender, morbidity (measured by diagnoses and in outpatient care partly by drug codes) and reduced earning capacity (until 2020). Since the year 2021, the regional over- and undercompensations are adjusted by up to ten regional variables. It is partly a prospective model as risk adjusters are from year *t* − 1 to predict spending in year *t*; age and gender variables are from year *t*. In addition, there are special models for sick leave payments and for insured residing abroad. For our estimations, we used the entire dataset and did not exclude any observations.

Most data used in our analysis are not publicly available. The German regulator (Federal Office for Social Security) performed the calculations, using data supplied by the sickness funds to the regulator. Insurers report their number and risk structure of enrollees, premium income, medical claim spending, subsidies from risk-equalization, and administrative costs. Administrative costs are divided by costs for staff (subdivided in a number of categories, for example for members of the board of directors, for old age pension programs, for regular salary, subsidies for staff canteen), for health campaigns, marketing, expenses for the offices, buying and maintaining cars etc. For our analysis, we used the total sum of administrative expenses.

## Appendix 4: The Netherlands

The following summary of the Dutch health insurance system draws from Douven et al. [16], Vektis [17] and Withagen-Koster et al. [11]. In 2006, by the introduction of a new Health Insurance Act, the Dutch government implemented a system of managed competition, along the lines of Enthoven [18], for the entire population. Former sickness funds and former private indemnity insurers are allowed to compete for providing basic health insurance to all Dutch citizens. To preserve universal access and maintain equity the government implemented mandatory insurance for a standardized basic benefit package, open enrollment, and a risk-equalization system. The basic benefit package covers about 44% of total health care costs, and includes the bulk of essential medical care, medications, medical aids, some physiotherapy and dental care.

The insurance market has significantly concentrated over the years. In 2006, basic health insurance was offered by 33 health insurers, but this number decreased to 24 insurers within 11 holdings in 2019. The four largest holdings together have a market share of more than 80%. There exists a mandatory deductible and health insurers can offer voluntary deductibles. Most large insurers offer voluntary supplemental health insurance, which is offered in a less regulated for-profit market. Some holdings also offer other types of insurance such as life or home insurance.

45% of the basic benefit package is funded by a community-rated premium and 50% by an employer-based income-related premium. The remaining 5% are government subsidies, for children under 18 as they do not have to pay a community-rated premium. People with low income receive compensation to help them pay their community-rated premium. Enrollees who still fail to pay their community-rated premium for 6 consecutive months are transferred from the insurer to the public National Administration Office.

The large majority of the insurers are not-for-profit. The reserve requirements for insurers have to follow the European Solvency II Directive. Insurers may (and actually do) use their excessive reserves, where appropriate, to lower their community-rated premium. In such cases, the residual loading fee of an insurer might even become negative.

The Dutch risk-equalization model has been continuously improved since 1991 and predicts medical spending (and not the loading fee), using individual risk characteristics like age, gender, region, socioeconomic status, source of income, and health indicators. The latter include several classifications related to morbidity, such as pharmacy-based cost groups, diagnoses-based cost groups, multiple-year high cost groups and durable medical equipment cost groups.

The data we use in our analysis are obtained from the Dutch government and contains information from ten insurance holdings until 2017 and nine holdings for 2018 and 2019. We have no information about one holding that entered in 2018 and one holding that entered in 2019. Both holdings had a relatively small population. It is mandatory for insurers to provide information to the regulator, such as the number of enrollees, premium income, medical claim spending, contributions to or from risk-equalization, and administrative costs. Administrative costs are divided into various subcategories, that changed over the years, such as claims handling costs, not risk-adjusted health care costs, advertisement costs, acquisition costs and other administrative costs. The data are only available at the holding level for the basic health insurance and not for supplemental or other types of insurance. For our estimations, we used the entire dataset and did not exclude any observations.

## Appendix 5: Switzerland

The following summary of the Swiss health insurance system draws heavily from Schmid et al. [19]. Historically a Bismarck system, the Swiss health care system switched to the managed competition model envisioned by Enthoven [18] by implementing a major reform in 1996. The reform introduced a standardized benefit package, open enrollment, and an insurance mandate so that insurers no longer face costs for underwriting. While a significant portion of health care financing still comes from taxes and out-of-pocket contributions, mandatory health insurance covers the largest fraction of total health care spending (about 38%).

Every Swiss resident must choose a health plan provided by competing private, not-for-profit insurers. The insurance market has significantly concentrated over the years with currently 51 insurers (1996: 159), with the 4 biggest covering more than half of the market. Of the 51 insurers only 38 are independent entities. Similar to the Netherlands, an insurer is allowed to establish carriers in a holding structure [20]. Most large insurers offer voluntary supplemental health insurance, which is offered in a less regulated for-profit market. Some insurers additionally offer other types of insurance (e.g., home, travel, life).

The premium for the basic, mandatory plan is on an individual level and not tied to employment or income but collected separately through a monthly bill. Therefore, premium collection is a major component of administrative costs for Swiss insurers as many customers fail to pay in time. Premiums are community-rated at the level of the region of residence and age (with discounts for children and young adults). Discounts are granted if the customer chooses a higher deductible than the default option or if the default free access to every provider is restricted. Excess premium income needs to be put into the reserves and can be used to lower the premium. Similarly, if medical spending turns out to be larger than premium income, the residual loading fee might be negative and needs to be covered by the reserves.

Unlike in most other countries, risk-equalization in Switzerland is not based on prior diagnoses. Before 2017, only canton, age, sex, and prior hospitalization were used as risk adjustors. From 2017 to 2019, a drug threshold was included. Starting in 2020, this threshold was replaced by Pharmaceutical Cost Groups (PCG). Children have always been excluded from risk-equalization. Another important difference to other countries is that the payments related to risk-equalization are reallocated between insurers so that part of the premium income is redistributed from insurers with below average risks to insurers with above average risks. Risk-equalization is performed on medical spending only, (and not on the loading fee).

The data used in our analysis are publicly available from the regulator at www.bag.admin.ch/kvstat. Insurers need to report their number of enrollees, premium income, medical claim spending, contributions to or from risk-equalization, and administrative costs. The breakdown of administrative costs into subcategories is very limited. The main administrative activities of insurers include processing claims, premium collection and customer service, while others play a minor role, e.g., purchasing care as the benefit package is standardized and prices are collectively bargained by associations. Insurers need to report administrative costs on the carrier level and not on the holding level. The data are only available for the basic health insurance and not for supplemental health insurance. For our estimations, we did not exclude any observations but used the entire dataset.

## Appendix 6: Marketplaces in the United States

The following summary of the US health insurance system draws from Layton, Montz, and Shepard [21] and Cox et al. [22]. The US health insurance market includes both public (e.g., Medicaid and Medicare) and private markets (e.g., employer-sponsored insurance). According to the US Census Bureau,^15^ in 2019, Medicaid and Medicare covered 17.2% and 18.1% of Americans, respectively. In the private market, employment-based insurance plans covered 56.4% of Americans, and 10.2% purchased their insurance plans themselves.

The private market could be further split into 3 segments by the type of consumers—individual, small-group (50 full-time employees or fewer), and large-group (more than 50 full-time employees). The first two segments are very similar in terms of regulations and market settings; but are distinct in plan generosity, premium level, and risk per purchase. The latter one, the large-group segment, is very different. For example, a large employer itself has a relatively stable average risk level. A large employer could also bargain with an insurer on plan benefits design and premiums, while an individual or a small business owner rarely has such power.

The private health insurance market in the US has been changed a lot under the Affordable Care Act (the ACA), starting from 2011. Some ACA provisions directly target premiums. Under the ACA, there is community rating and premiums are no longer allowed to vary based on individual features except age and tobacco use status. For example, insurers cannot deny coverage or increase premiums for consumers with pre-existing conditions. Another important provision for insurance plan design is the list of essential health benefits, ranging from inpatient, outpatient, to preventive, to mental health services. Some other provisions may also affect the private market, such as the Medicaid expansion, which expands the public coverage to many previously ineligible people; the individual mandate, which requires individuals to buy insurance plans or pay a penalty; and the premium subsidy and cost-sharing subsidy for low-income enrollees. All those provisions affect the risk pool of the private market.

In 2014, the ACA Health Insurance Exchange Marketplace was introduced to provide a platform for individuals or small groups to purchase health insurance plans and to ease the purchase process. Each year, about 11.4 million Americans obtain insurance coverage from the Marketplace.^16^ All the insurers could participate in the Marketplace as long as they provide one basic (silver) plan in the market. Following the opening of the Marketplace, to mitigate the risk selection problem and stabilize premiums, the ACA also included risk adjustment, reinsurance and risk corridors. Risk adjustment is a permanent program, which uses the HHS-HCC risk score to predict enrollees’ medical spending and transfer from low-risk insurers to high-risk insurers within a state year [23]. The other two programs were temporary, in place from 2014–2016. The reinsurance program pays plans if enrollees’ actual (rather than predicted) cost exceeds a threshold. That is a net flow into the Marketplace. The risk corridor collects funds from plans with low actual claims and transfers them to plans with high actual claims.

The data used in this paper stem from the medical loss ratio reports, which are publicly available from the Center for Consumer Information and Insurance Oversight (CCIIO) https://www.cms.gov/CCIIO/Resources/Data-Resources/mlr. The administrative costs include agents and brokers’ fees and commissions, cost containment expenses not included in quality improvement expenses, direct sales salaries and benefits, fines and penalties of regulatory authorities, taxes and assessments, federal and state taxes and licensing or regulatory fees, other general and administrative expenses, and other claims adjustment expenses. The data are observed at the insurer-market level.

For the estimations in the paper, the data were cleaned in several ways. In both segments, we removed outliers by dropping in the individual segment small insurers with a population size of less than 1000 enrollees, and in the small group market insurers with less than 2000 enrollees. Moreover, in both markets, we dropped observations that belong to the 1% outliers related to mean administrative costs per life year and the 1% outliers related to mean health care expenditures per life year, and in the small group market 1% outliers related to the risk-score. Also, we excluded five insurers that changed their non-for-profit status during 2017–2019, as their administrative costs may be strongly related to this change.

## Footnotes

1 Without premium regulation the goal of risk-equalization would be to make health insurance affordable [8].

2 Except for Germany all data is publicly available and can be retrieved from the authors upon request.

3 This is the premium per life year that an insurer receives via the out-of-pocket premium from the enrollee and includes subsidies and the equalization payments from/to the regulator.

4 In the US, the medical loss ratio (MLR) is a measure of the percentage of premium dollars that a health plan spends on medical claims. In that sense, it is equal to 1—loading fee. Under the Affordable Care Act (ACA) rules, insurers must remit a rebate if the MLR falls below 80%. The ACA regulator however uses a slightly different rule for the MLR. For example, to increase comparability with the other countries, we included spending by insurers on quality improvements in the loading fee, while the ACA regulator adds this term to the MLR. Another difference is that when testing for the 80% threshold, the ACA regulator adjusts MLRs of insurers to control for population size. Therefore, our MLRs are likely to be somewhat lower than the ratios used by the ACA regulator.

5 First, the quality of the risk-equalization system, i.e. mainly the number of risk adjusters, differs across countries which feeds through to the risk-score. Second, in some countries, premiums charged to specific groups of individuals may differ. A measurement error occurs when there is skewness in the distribution for these specific groups of enrollees across insurers. See also “Appendix 1”.

6 Future work should study whether it is possible for the Netherlands to obtain information at the insurer level instead of the holding level, which would raise the number of observations considerably.

7 In countries where insurers are not allowed to charge an out-of-pocket premium from their enrollees (e.g. Colombia, Israel, and the Russian Federation) a compensation for the loading fee might be included in the risk-equalization [24–26]. For example, in Colombia the loading fee is 8–10% of the equalization payment insurers receive [24, p. 287].

8 This prediction is the starting point for determining the equalization payments. The ultimate payments to insurers depend on the modality of payment flows in a country. In practice, each country uses different payment modalities [2, 8].

9 Thus $${{{\widehat{\delta }}_{it}= {\widehat{\beta }}_{0}+\widehat{\beta }}_{2}{\mathrm{popsize}}_{it}+ {\widehat{\beta }}_{3}\left(\frac{1}{{ \mathrm{popsize}}_{it}}\right)+ \widehat{\alpha }}_{i}+{\widehat{\gamma }}_{t}$$.

10 Note that these percentages would be smaller for all countries, and for most countries much smaller, if we used the estimates for the ex-post risk-scores from Table 3 in “Appendix 1”.

11 The US regulator considers “14% of the average premium in the market” to reflect the proportion of the administrative costs that do not vary with medical claims.

12 To obtain an estimate we use the numbers in Tables 1 and 2. For the individual market the administrative costs that do not vary with medical claims are equal to 849.4–509.6 = 339.8 = 0.061*$\overline{P}$, and for the small group market we find 824.8–471.4 = 353.4 = 0.065*$\overline{P}$ which implies that about 6% of the administrative costs do not vary with medical claims. A similar exercise with the ex-post risk score implies that about 15% of the administrative costs do not vary with medical claims. Note that we assume here that 100% of the residual loading fee is included in the risk-equalization.

13 Besides an insurer’s risk-score there might be other characteristics of enrollees that should be taken into account, such as the probability that an enrollee ends up in payment arrear.

14 In the US, the regulator only mentions administrative costs (see Sect. 4.2) which suggests that 100% of the residual loading fee in the US Marketplaces is included in the risk-equalization. The size of the residual loading fee is about 3.2% of total premiums in the individual market and 5.9% in the group market in 2017–2019. In Germany, the residual loading fee is not included in the risk-equalization but the question here is less relevant as the amount is much smaller, about 0.5% of total premiums in 2014–2019.

## Code availability

See above, all computations and coding were performed with the software program STATA or SAS. The code can be retrieved from the corresponding author upon request.
